# Downregulation of PLK4 expression induces apoptosis and G0/G1‐phase cell cycle arrest in keloid fibroblasts

**DOI:** 10.1111/cpr.13271

**Published:** 2022-06-07

**Authors:** Ru‐Lin Huang, Chuanqi Liu, Rao Fu, Yuxin Yan, Jing Yang, Xinggang Wang, Qingfeng Li

**Affiliations:** ^1^ Department of Plastic and Reconstructive Surgery, Shanghai Ninth People's Hospital Shanghai Jiao Tong University School of Medicine Shanghai China; ^2^ Department of Plastic and Burn Surgery, West China Hospital Sichuan University Chengdu China; ^3^ Department of Assisted Reproduction, Shanghai Ninth People's Hospital Shanghai Jiao Tong University School of Medicine Shanghai China

## Abstract

**Objectives:**

Keloids are benign fibroproliferative tumors that display many cancer‐like characteristics, such as progressive uncontrolled growth, lack of spontaneous regression, and extremely high rates of recurrence. Polo‐like kinase 4 (PLK4) was recently identified as a master regulator of centriole replication, and its aberrant expression is closely associated with tumorigenesis. This study aimed to investigate the expression and biological role of PLK4 in the pathogenesis of keloids.

**Materials and Methods:**

We evaluated the expression of PLK4 in keloids and adjacent normal skin tissue samples. Then, we established PLK4 knockdown and overexpression cell lines in keloid fibroblasts (KFs) and normal skin fibroblasts (NFs), respectively, to investigate the roles of PLK4 in the regulation of proliferation, migration, invasion, apoptosis, and cell cycle in KFs. Centrinone B (Cen‐B), a highly selective PLK4 inhibitor, was used to inhibit PLK4 activity in KFs to evaluate the therapeutic effect on KFs.

**Results:**

We discovered that PLK4 was overexpressed in keloid dermal samples and KFs compared with adjacent normal skin samples and NFs derived from the same patients. High PLK4 expression was positively associated with the proliferation, migration, and invasion of KFs. Furthermore, knockdown of PLK4 expression or inhibition of PLK4 activity by Cen‐B suppressed KF growth, induced KF apoptosis via the caspase‐9/3 pathway, and induced cell cycle arrest at the G0/G1 phase in vitro.

**Conclusions:**

These findings demonstrate that PLK4 is a critical regulator of KF proliferation, migration, and invasion, and thus, Cen‐B is a promising candidate drug for keloid treatment.

## Graphical abstract

Keloids are benign fibroproliferative tumors that display many cancer‐like characteristics, such as progressive uncontrolled growth, lack of spontaneous regression, and extremely high rates of recurrence. Polo‐like kinase 4 (PLK4) was recently identified as a master regulator of centriole replication, and its aberrant expression is closely associated with tumorigenesis. This study aimed to investigate the expression and biological role of PLK4 in the pathogenesis of keloids. Here, we discovered that PLK4 is a potential target for the treatment of keloids. PLK4 was overexpressed in keloid dermal samples and keloid fibroblasts (KFs) compared with adjacent normal skin samples and normal skin fibroblasts derived from the same patients. High PLK4 expression was positively associated with the proliferation, migration, and invasion of KFs. Furthermore, knockdown of PLK4 expression or inhibition of PLK4 activity by a highly selective inhibitor, centrinone B (Cen‐B), suppressed KF growth, induced KF apoptosis via the caspase‐9/3 pathway, and induced cell cycle arrest at the G0/G1 phase via the p53/p21/Cyclin D1 pathway in vitro. These findings demonstrate that PLK4 is a critical regulator of KF proliferation, migration, and invasion, and thus, Cen‐B is a promising candidate drug for keloid treatment.

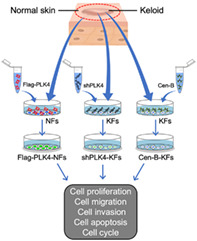

## 1 INTRODUCTION

Keloids are benign fibroproliferative reticular dermal tumors of unknown etiopathogenesis that can occur following any dermal injury, leading to an exophytic protuberant outgrowth that invades the adjacent normal skin beyond the original wound boundary.^1^, ^2^, ^3^ Keloids are uniquely characterized by their aggressiveness, persistence, and progressive perilesional expansile behaviors. Due to their continued growth and lack of spontaneous regression, patients usually experience intense pain and pruritus, especially for keloid contractures located near the joints, which may lead to serious dysfunction and thus affect patients' physiological and psychological health.

Several common therapeutic approaches have been used for keloid prevention and treatment, and the main treatments include surgical excision in combination with local radiotherapy, steroid injections, and laser treatment. However, these strategies have been proven to be largely ineffective, and keloids can easily recur.^4^, ^5^, ^6^ Therefore, novel therapeutic interventions for keloids are still needed. Clinically, the features of keloids are consistent with those of nonmalignant dermal tumors due to the excessive overproduction of collagen, invasive outgrowth, and lack of metastatic potential.^7^ Biologically, these features are mainly supported by hyperproliferative fibroblasts, which exhibit increased proliferation and decreased apoptosis, leading to the invasion of activated fibroblasts into the adjacent tissue.^8^, ^9^ This phenomenon suggests that targeting the factors regulating keloid fibroblast (KF) proliferation or apoptosis may be a therapeutic strategy for keloid treatment.

Polo‐like kinase 4 (PLK4) is a serine/threonine‐protein kinase that functions as a master regulator of the cell cycle and centriole duplication.^10^ The PLK4 enzyme is expressed at low levels in proliferating tissues and has pleiotropic functions in processes related to mitotic progression, including cytokinesis, cell mobility, and DNA damage repair.^11^, ^12^ Increasing evidence indicates that PLK4 is aberrantly expressed in patient‐derived tumor samples, including colorectal cancer,^13^, ^14^ breast cancer,^15^, ^16^ bladder cancer,^17^ and melanoma,^18^ but is also expressed at low levels in proliferative tissues.^19^, ^20^, ^21^ However, its expression level differs among cancers and proliferative tissues. Moreover, high expression of PLK4 endows cancer cells with invasive and metastatic abilities.^22^, ^23^ Blockade of PLK4 by selective small‐molecule inhibitors or RNA interference impairs centriole duplication, enhances genomic instability, and ultimately causes mitotic defects and cell death.^24^ In addition, inhibition of PLK4 by small‐molecule inhibitors, including CFI‐400945, YLT‐11, and centrinone B (Cen‐B), could suppress tumor growth^25^, ^26^ or increase sensitivity to radiation^15^, ^27^ and chemotherapy.^28^, ^29^ Due to its key role in tumor growth and development, PLK4 is considered one of the most promising therapeutic targets in cancer.

Keloids bear a strong resemblance to tumors, especially regarding their clinical presentation. Among all the similarities, the most compelling parallels between keloids and tumors are their shared cellular bioenergetics, epigenetic methylation profiles, epithelial‐to‐mesenchymal transition phenotypes, and tumor‐related biomarker profiles.^1^ Considering the tumor‐like characteristics of keloids, we hypothesized that PLK4 is overexpressed in KFs, which results in the uncontrolled growth and invasiveness of keloids via apoptosis and cell cycle regulation. In the present study, we investigated the expression patterns of PLK4 in keloids and normal skin and the role of PLK4 in apoptosis and cell cycle regulation in KFs for the first time. We confirmed that PLK4 expression was significantly elevated in keloid dermis samples compared to normal skin samples and that this upregulation was associated with the enhanced proliferation, migration, and invasion capacities of KFs. Downregulation of PLK4 expression in KFs enhanced apoptosis via the caspase‐9/3 pathway and may induce cell cycle arrest at the G0/G1 phase via the p53/p21/Cyclin D1 pathway. Furthermore, Cen‐B treatment and PLK4 downregulation exerted consistent effects and induced molecular pathway changes. These findings provide a potential treatment strategy for keloids based on targeting PLK4 with Cen‐B.

## 2 RESULTS

### 2.1 PLK4 expression is markedly elevated in human keloid superficial dermis samples and KFs

**FIGURE 1:**
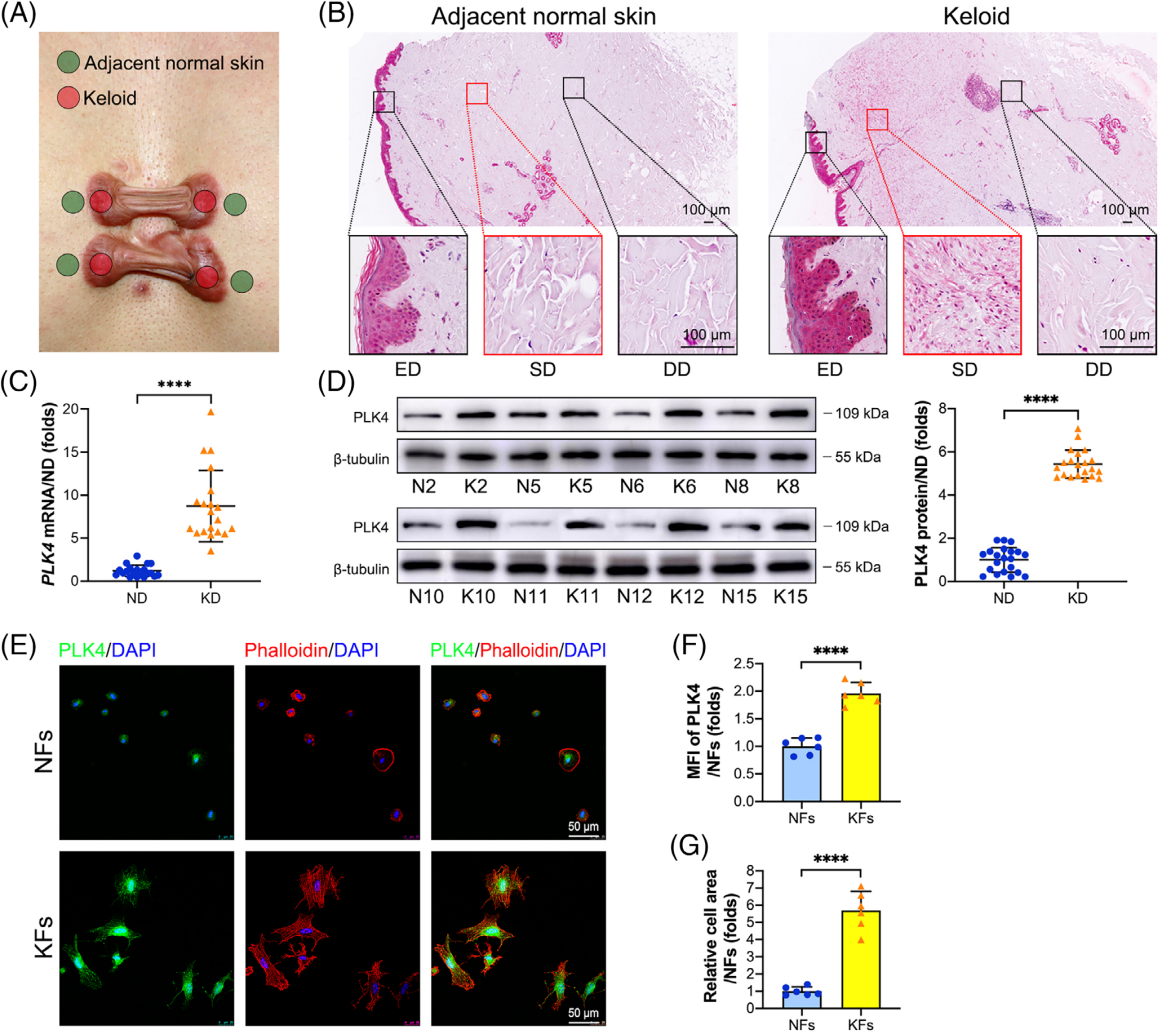
PLK4 expression is markedly elevated in keloid superficial dermal samples and KFs. (A) Illustration of the lesional sites of keloids and adjacent normal skin samples in this study. (B) Representative images of immunohistochemistry staining of PLK4 in keloid and matched adjacent normal skin tissue samples (*n* = 6 donors). (C) Real‐time PCR analysis of the *PLK4* mRNA levels in keloid dermal and matched adjacent normal skin dermal samples (*n* = 21 donors; ****p < 0.0001, compared with ND). (D) Representative images of western blot analysis of PLK4 protein expression in keloid dermal and matched adjacent normal skin dermal samples (*n* = 21 donors; ****p < 0.0001, compared with ND). (E) Primary KFs and NFs from the same donors were seeded in confocal dishes at a density of 1.6 × 10^5^ cells/cm^2^ and cultured for 8 h. Representative images of immunofluorescence staining of PLK4 (green), phalloidin (red), and 4',6‐diamidino‐2‐phenylindole (DAPI) (blue) (*n* = 6 donors). (F) Quantitative analysis of the relative MFI of PLK4 (*n* = 6 donors; ****p < 0.0001, compared with NFs). (G) Quantitative analysis of the relative cell spreading area using ImageJ (*n* = 6 donors; ****p < 0.0001, compared with NFs). DD, deep dermis; ED, epidermis; KD, keloid dermis; KFs, keloid fibroblasts; MFI, mean fluorescence intensity; ND, normal skin dermis; NFs, normal skin fibroblasts; PLK4, Polo‐like kinase 4; SD, superficial dermis.

We first evaluated the expression of PLK4 in keloids and adjacent normal skin tissue samples from patients who were clinically diagnosed with keloids (Figure 1A). As shown in Figure 1B, PLK4 was expressed in both keloids and normal skin epidermal tissue. However, PLK4 expression was markedly elevated in the keloid dermis, especially the superficial dermis, compared with adjacent normal skin (Figure 1B). This result was further confirmed by real‐time polymerase chain reaction (PCR) and western blot analysis of all 21 pairs of collected samples. Real‐time PCR analysis revealed that *PLK4* mRNA expression in keloid dermal tissue was approximately 7.4‐fold higher than that in the adjacent normal skin dermis (Figure 1C). Furthermore, western blot analysis demonstrated that PLK4 protein expression in keloid dermal tissue was 5.4‐fold higher than that in the adjacent normal skin dermis (Figure 1D). The expression of PLK4 was further explored in fibroblasts isolated from six pairs of keloid and adjacent normal skin dermal tissue samples. To minimize the potential influence of in vitro culture on PLK4 expression and allow sufficient cell adherence to the confocal dishes, the primary fibroblasts were cultured for 8 h and then double‐stained with PLK4 and phalloidin. PLK4 was expressed in the nucleoli and cytoplasm of both KFs and normal skin fibroblasts (NFs) (Figure 1E). However, after 8 h of culture, the relative mean fluorescence intensity (MFI) of PLK4 in KFs was significantly higher than that in NFs (Figure 1F). Furthermore, the KFs spread over a larger area than the NFs (Figure 1G). Interestingly, immunostaining of γ‐tubulin foci indicated that there was no significant difference between centrosome number distributions between KFs and NFs from the same donors (Figure S2). Taken together, these results indicated that PLK4 expression is elevated in keloid dermal samples and KFs.

### 2.2 Knockdown of PLK4 inhibits the proliferation, migration, and invasion of KFs

**FIGURE 2:**
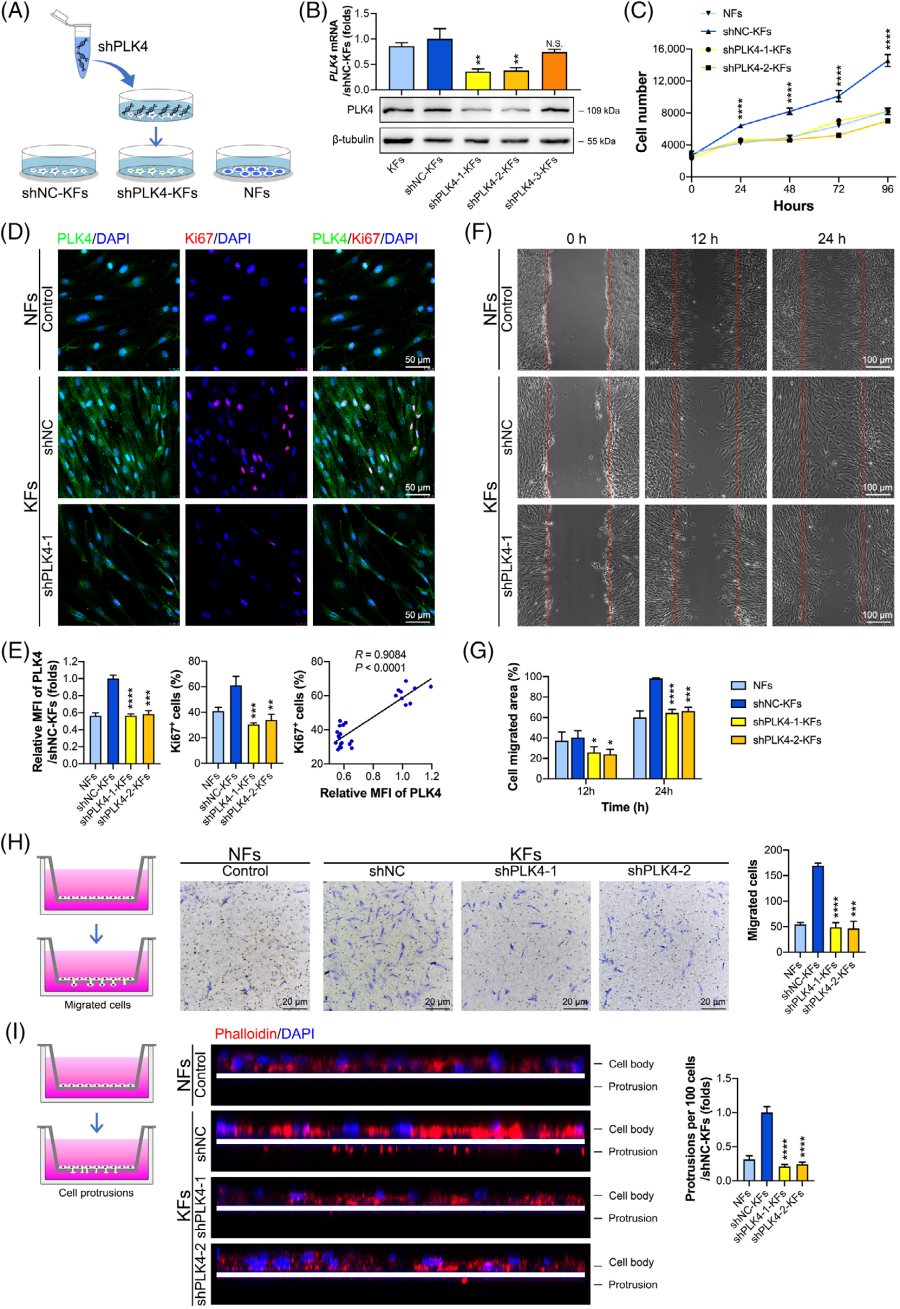
Knockdown of PLK4 inhibits KF proliferation, migration, and invasion. (A) Schematic diagram of the generation of shNC‐KF and shPLK4‐KF cell lines by transfection of KFs with vector (shNC‐KFs) and shPLK4 lentiviruses (shPLK4‐1‐KFs, shPLK4‐2‐KFs, and shPLK4‐3‐KFs), respectively. (B) Real‐time PCR (*above*) and western blot (*below*) analysis of PLK4 expression in KFs, shNC‐KFs, shPLK4‐1‐KFs, shPLK4‐2‐KFs, and shPLK4‐3‐KFs at 7 days post‐transfection (*n* = 3 donors; **p < 0.01, compared with shNC‐KFs). (C) CCK‐8 assays of shNC‐KFs, shPLK4‐1‐KFs, shPLK4‐2‐KFs, and NFs were performed at 0, 24, 48, 72, and 96 h (*n* = 3 donors; ****p < 0.0001, compared with shNC‐KFs). (D) Cell lines were seeded in confocal dishes at a density of 1.6 × 10^5^ cells/cm^2^ and cultured for 48 h. Representative images of immunofluorescence staining of PLK4 and Ki67 in shNC‐KFs, shPLK4‐1‐KFs, and NFs at 7 days post‐transfection (*n* = 3 donors). (E) *Left*, Quantitative analysis of the relative MFI of PLK4 expression (*n* = 3 donors; ***p < 0.001, ****p < 0.0001, compared with shNC‐KFs). *Middle*, Percentage of Ki67^+^ cells (*n* = 3 donors; **p < 0.01, ***p < 0.001, compared with shNC‐KFs). *Right*, The correlation between the relative MFI of PLK4 and Ki67^+^ cell percentages in KFs was determined by Pearson correlation coefficient analysis (*R* = 0.9084; *p* < 0.0001). (F) Scratch wound healing assays of shNC‐KFs, shPLK4‐1‐KFs, and NFs were performed at 7 days post‐transfection, and representative images of the healed scratches at 0, 12, and 24 h are shown (*n* = 3 donors). (G) Quantitative analysis of the cell migration area (*n* = 3 donors; *p < 0.05, ***p < 0.001, ****p < 0.0001, compared with shNC‐KFs). (H) *Left*, Schematic diagram of the Transwell invasion assay. *Middle*, cell invasion assays of shNC‐KFs, shPLK4‐1‐KFs, shPLK4‐2‐KFs, and NFs on 8‐μm pore size Transwell filters were performed; representative images of the migrated cells are shown. *Right*, Quantitative analysis of the number of migrated cells (*n* = 3 donors; ***p < 0.001, ****p < 0.0001, compared with shNC‐KFs). (I) *Left*, Schematic diagram of the cell protrusion assays. *Middle*, Cell protrusion assays of shNC‐KFs, shPLK4‐1‐KFs, shPLK4‐2‐KFs, and NFs on 1‐μm pore size Millicell inserts were performed by dual staining with phalloidin (red) and Hoechst (blue). Representative confocal Z‐section images of the cells are shown, and the white line marks the position of the filter. Cells were separated into the cell body and protrusion fractions. *Right*, Quantitative analysis of the number of protrusions per 100 cells (*n* = 3 donors; ****p < 0.0001, compared with shNC‐KFs). CCK‐8, Cell Counting Kit‐8; KFs, keloid fibroblasts; NFs, normal skin fibroblasts; PLK4, Polo‐like kinase 4; shRNAs, short hairpin RNAs; shNC, negative control shRNA.

To investigate the biological function of PLK4 in keloids, we used three lentivirus‐mediated short hairpin RNAs (shRNAs) and the negative control (NC) shRNA (shNC) to knockdown PLK4 expression in KFs. Consequently, we established three PLK4 knockdown KF cell lines (shPLK4‐1‐KFs, shPLK4‐2‐KFs, and shPLK4‐3‐KFs) and their counterpart negative control KF cell line (shNC‐KFs) (Figure 2A). Then, the mRNA and protein levels of PLK4 expression in KFs, shNC‐KFs, shPLK4‐1‐KFs, shPLK4‐2‐KFs, and shPLK4‐3‐KFs were examined by real‐time PCR and western blot analyses. The results demonstrated that shPLK4‐1 lentivirus transfection decreased the *PLK4* mRNA levels by approximately 64.3% and the protein levels by approximately 65.8% compared to those in the shNC lentivirus‐transfected KFs. Similarly, shPLK4‐2 lentivirus transfection decreased the *PLK4* mRNA levels by approximately 62.1% and the protein levels by approximately 60.2% compared to those in shNC lentivirus‐transfected KFs. However, shPLK4‐3 and shNC lentivirus transfection did not influence PLK4 expression at either the mRNA or protein level (Figure 2B). These data confirmed the successful establishment of PLK4 knockdown cell lines (shPLK4‐1‐KFs and shPLK4‐2‐KFs). Then, the effects of PLK4 knockdown on the proliferation, migration, and invasion of these cells were assessed. The Cell Counting Kit‐8 (CCK‐8) assay showed that knockdown of PLK4 decreased the proliferative capacity of KFs (shPLK4‐1‐KFs and shPLK4‐2‐KFs) compared with that of shNC‐KFs, comparable to NFs (Figure 2C). To further investigate the relevance of PLK4 expression and proliferation, we performed immunofluorescence dual staining of PLK4 and Ki67 (an indicator of proliferation) (Figures 2D and S1A). The MFI of PLK4 in shPLK4‐1‐KFs and shPLK4‐2‐KFs decreased by 43.6% and 41.6%, respectively, compared with that in shNC‐KFs, which was comparable to that in NFs (Figure 2E, left). Moreover, the percentage of Ki67^+^ cells decreased from 61.0% in shNC‐KFs to 30.4% in shPLK4‐1‐KFs and 33.9% in shPLK4‐2‐KFs (Figure 2E, middle). According to Pearson correlation coefficient analysis, a positive correlation was observed between PLK4 expression and the percentage of Ki67^+^ cells (*R* = 0.9084, *p* < 0.001) among KFs (Figure 2E, right). Cell migration was assessed by scratch wound healing assays (Figures 2F and S1B), revealing that the migration of shPLK4‐1‐KFs and shPLK4‐2‐KFs significantly decreased in a time‐dependent manner compared with that of shNC‐KFs, especially at 24 h (Figure 2G). Cell invasion was investigated by Transwell invasion assays (Figure 2H) and cell protrusion assays (Figure 2I). Knockdown of PLK4 by transfection of shPLK4‐1 and shPLK4‐2 decreased the number of migrated KFs in the Transwell invasion assay (Figure 2H). The cell protrusion assay was used to measure invasiveness by quantifying the number of cell protrusions invading through a porous mesh. As shown in Figure 2I, control KFs formed numerous protrusions after 10 h of in vitro culture, while KFs with PLK4 knockdown showed a significantly suppressed protrusion formation ability. Collectively, these data indicated that knockdown of PLK4 in KFs inhibits cell proliferation, migration, and invasion.

### 2.3 Overexpression of PLK4 promotes the proliferation, migration, and invasion of NFs

**FIGURE 3:**
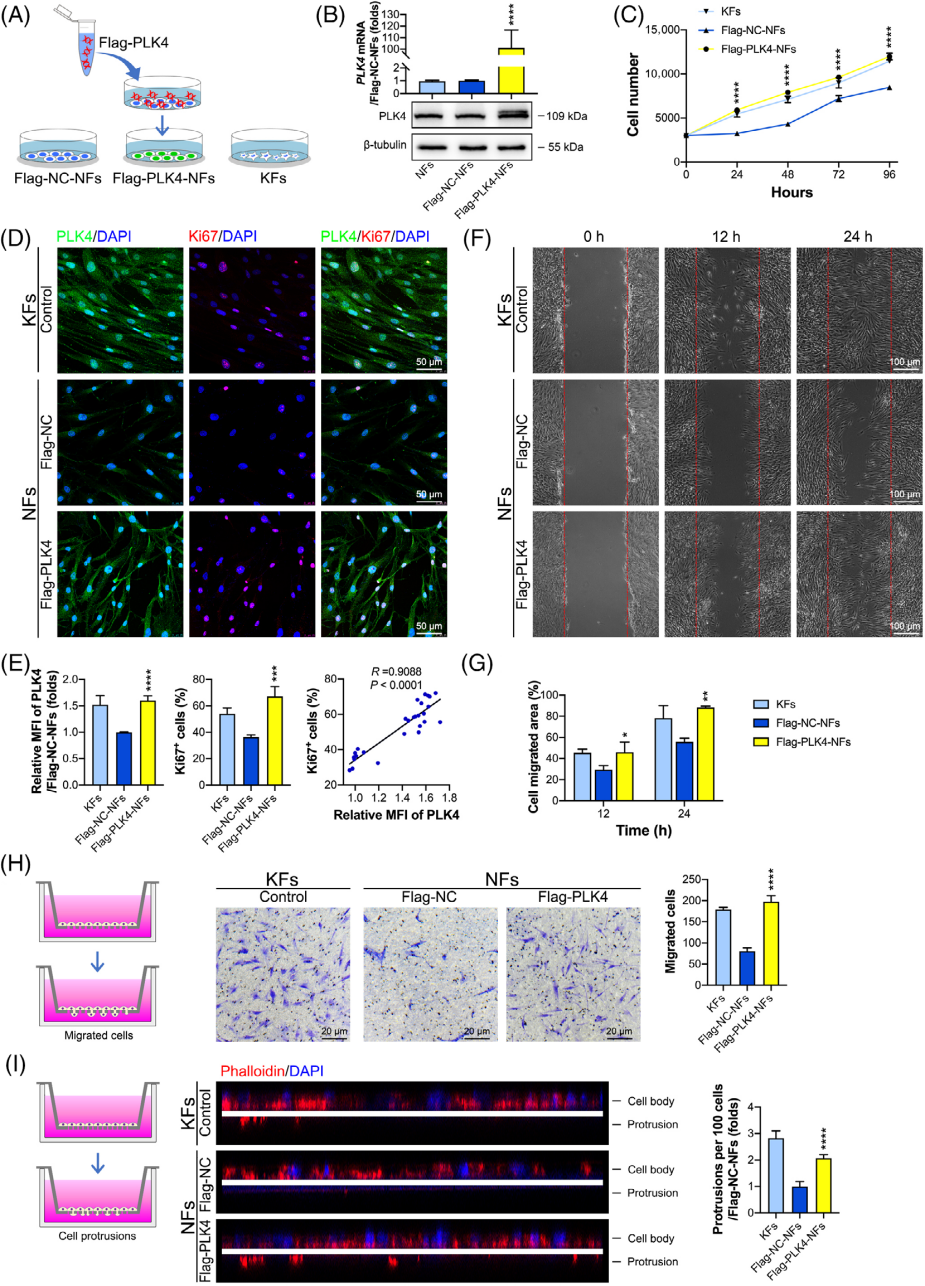
Overexpression of PLK4 promotes NF proliferation, migration, and invasion. (A) Schematic diagram of the generation of the Flag‐NC‐NF and Flag‐PLK4‐NF cell lines by the transfection of NFs with vector (Flag‐NC‐NFs) and Flag‐PLK4 lentiviruses (Flag‐PLK4‐NFs), respectively. (B) Real‐time PCR (*above*) and western blot (*below*) analysis of the PLK4 expression in NFs, Flag‐NC‐NFs, and Flag‐PLK4‐NFs at 7 days post‐transfection (*n* = 3 donors; ****p < 0.0001, compared with Flag‐NC‐NFs). (C) The proliferation of Flag‐NC‐NFs, Flag‐PLK4‐NFs, and KFs was assessed by the CCK‐8 assay at 0, 24, 48, 72, and 96 h (*n* = 3 donors; ****p < 0.0001, compared with Flag‐NC‐NFs). (D) Cell lines were seeded in confocal dishes at a density of 1.6 × 10^5^ cells/cm^2^ and cultured for 48 h. Representative images of immunofluorescence staining of PLK4 and Ki67 in Flag‐NC‐NFs, Flag‐PLK4‐NFs, and KFs (*n* = 3 donors). (E) *Left*, Quantitative analysis of the relative MFI of PLK4 expression (*n* = 3 donors; ****p < 0.0001, compared with Flag‐NC‐NFs). *Middle*, Percentage of Ki67^+^ cells (*n* = 3 donors; ***p < 0.001, compared with the Flag‐NC‐NFs). *Right*, The correlation between the relative MFI of PLK4 and Ki67^+^ cell percentages in NFs was determined by Pearson correlation coefficient analysis (*R* = 0.9088; *p* < 0.0001). (F) Scratch wound healing assays of Flag‐NC‐NFs, Flag‐PLK4‐NFs, and KFs were performed, and representative images of the healed scratches at 0, 12, and 24 h are shown (*n* = 3 donors). (G) Quantitative analysis of the cell migration area (*n* = 3 donors; *p < 0.05, **p < 0.01, compared with Flag‐NC‐NFs). (H) *Left*, Schematic diagram of the Transwell invasion assays. *Middle*, cell invasion assays of Flag‐NC‐NFs, Flag‐PLK4‐NFs, and KFs were performed, and representative images of the migrated cells are shown. *Right*, Quantitative analysis of the number of migrated cells (*n* = 3 donors; ****p < 0.0001, compared with Flag‐NC‐NFs). (I) *Left*, schematic diagram of the cell protrusion assay. *Middle*, Cell protrusion assays of Flag‐NC‐NFs, Flag‐PLK4‐NFs, and KFs were performed by dual staining with phalloidin (red) and Hoechst (blue). Representative confocal Z‐section images of the cells are shown. The white line marks the position of the filter. Cells were separated into the cell body and protrusion fractions. *Right*, Quantitative analysis of the number of protrusions per 100 cells (*n* = 3 donors; ****p < 0.0001, compared with Flag‐NC‐NFs). CCK‐8, Cell Counting Kit‐8; KFs, keloid fibroblasts; NFs, normal skin fibroblasts; PLK4, Polo‐like kinase 4.

To further confirm that the high proliferation, migration, and invasion capacities of KFs were related to a high expression level of PLK4, NFs were transfected with lentivirus encoding Flag‐tagged PLK4 (Flag‐PLK4) and the negative control (Flag‐NC), establishing PLK4 overexpression NF cell line (Flag‐PLK4‐NFs) and its counterpart negative control NF cell line (Flag‐NC‐NFs) (Figure 3A). Real‐time PCR and western blot analyses confirmed that the mRNA and protein levels of PLK4 were increased in the Flag‐PLK4‐NFs. Specifically, the *PLK4* mRNA and protein levels of Flag‐PLK4‐NFs were approximately 101.1‐fold higher and 2.1‐fold higher than those in Flag‐NC lentivirus‐transfected NFs, while the transfection of NFs with Flag‐NC lentivirus did not affect PLK4 expression at the mRNA or protein levels (Figure 3B), indicating the successful establishment of the Flag‐PLK4‐NF and Flag‐NC‐NF cell lines. The CCK‐8 assay showed that overexpression of PLK4 in NFs significantly promoted their proliferation to a level comparable to that of KFs (Figure 3C). Similar results were obtained by immunofluorescence dual staining of PLK4 and Ki67 (Figure 3D). Overexpression of PLK4 in NFs increased the MFI of PLK4 to approximately 60.3% of that in Flag‐NC‐NFs (Figure 3F, left), while the percentage of Ki67^+^ cells increased from 36.4% in Flag‐NC‐NFs to 67.2% in Flag‐PLK4‐NFs (Figure 3F, middle). Pearson correlation coefficient analysis demonstrated a positive correlation between PLK4 expression and the percentage of Ki67^+^ cells (*R* = 0.9088, *p* < 0.001) in NFs (Figure 3E, right). These results further suggested that the expression level of PLK4 was associated with the proliferation capacity of fibroblasts. Similarly, the scratch wound healing assay (Figure 3F,G), Transwell invasion assay (Figure 3H), and cell protrusion assay (Figure 3I) showed that overexpression of PLK4 enhanced the migration and invasion capabilities of NFs.

### 2.4 High PLK4 expression inhibits KF apoptosis via the caspase‐9/3 pathway

**FIGURE 4:**
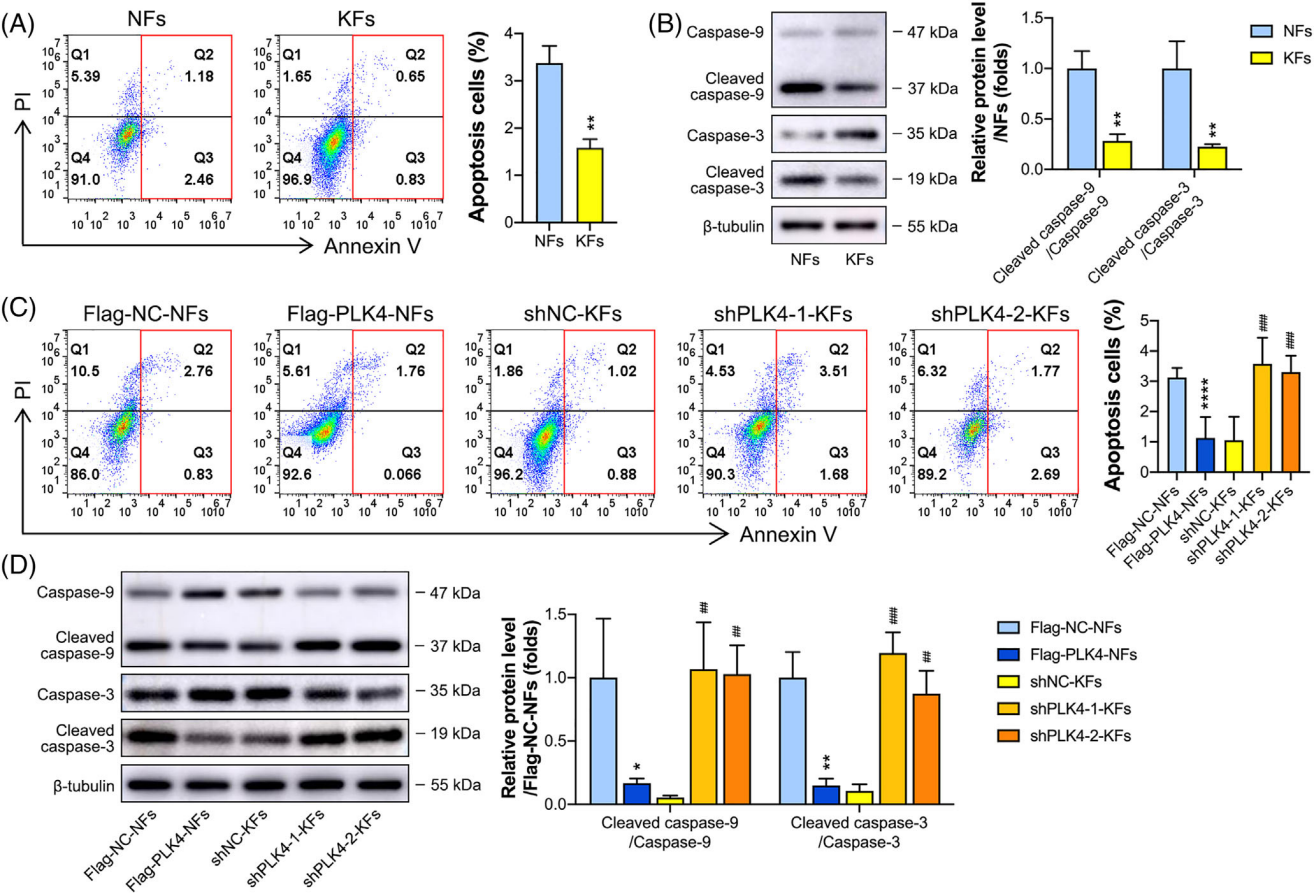
PLK4 inhibits KF apoptosis via the caspase‐9/3 pathway. (A) *Left*, Flow cytometry analysis of KF and NF apoptosis using annexin V/propidium (PI) dual labeling, and representative images are shown (*n* = 3 donors). *Right*, Quantitative analysis of apoptotic cells, including early phase (annexin V^+^ PI^−^) and late phase (annexin V^+^ PI^+^) apoptotic cells (*n* = 3 donors; **p < 0.01, compared with NFs). (B) *Left*, Western blot analysis of apoptosis‐related proteins, including caspase‐9, cleaved caspase‐9, caspase‐3, and cleaved caspase‐3 in NFs and KFs, and representative images are shown (*n* = 3 donors). *Right*, Quantitation of apoptosis‐related protein levels by densitometric analysis using ImageJ (*n* = 3 donors; **p < 0.01, compared with NFs). (C) *Left*, Flow cytometry analysis of apoptosis using annexin V/PI dual labeling in Flag‐NC‐NFs, Flag‐PLK4‐NFs, shNC‐KFs, shPLK4‐1‐KFs, and shPLK4‐2‐KFs, and representative images are shown (*n* = 3 donors). *Right*, Quantitative analysis of apoptotic cells (*n* = 3 donors; ****p < 0.0001, compared with Flag‐NC‐NFs; ###*p* < 0.001, compared with shNC‐KFs). (D) *Left*, Western blot analysis of caspase‐9, cleaved caspase‐9, caspase‐3, and cleaved caspase‐3 expression in Flag‐NC‐NFs, Flag‐PLK4‐NFs, shNC‐KFs, shPLK4‐1‐KFs, and shPLK4‐2‐KFs, and representative images are shown (*n* = 3 donors). *Right*, Quantitation of apoptosis‐related protein levels by densitometric analysis using ImageJ (n = 3 donors; *p < 0.05, **p < 0.01, compared with Flag‐NC‐NFs; ##*p* < 0.01, ###*p* < 0.001, compared with shNC‐KFs). KFs, keloid fibroblasts; NFs, normal skin fibroblasts; PLK4, Polo‐like kinase 4; shRNAs, short hairpin RNAs; shNC, negative control shRNA.

KFs reportedly exhibit a lower apoptotic rate than NFs,^30^ which was confirmed by flow cytometric analysis in this study. The percentage of apoptotic cells among NFs was 2.1‐fold higher than that among KFs (Figure 4A). To investigate the possible underlying mechanism of PLK4‐mediated cell apoptosis, the expression of apoptosis‐related proteins in KFs and NFs was detected by western blotting (Figure 4B). The results showed that KFs expressed higher levels of cleaved caspase‐9 (an initiator caspase) and cleaved caspase‐3 (an effector caspase) than NFs (Figure 4B,C), indicating that PLK4 may inhibit apoptosis by inhibiting the caspase‐9/3 pathway in KFs. To further confirm this hypothesis, we subjected the established PLK4 knockdown and overexpression cell lines (shNC‐KFs, shPLK4‐1‐KFs, shPLK4‐2‐KFs, Flag‐NC‐NFs, and Flag‐PLK4‐NFs) to cell apoptosis analysis by flow cytometry and western blotting. The flow cytometry results demonstrated that the percentages of apoptotic cells among the shPLK4‐1‐KFs and shPLK4‐2‐KFs were 3.4‐ and 3.1‐fold, respectively, higher than those among the shNC‐KFs. In contrast, the percentage of apoptotic Flag‐PLK4‐NFs was 63.8% lower than that of the Flag‐NC‐NFs (Figure 4C). Furthermore, PLK4 knockdown KFs exhibited lower levels of the cleavage products of caspase‐3 and caspase‐9 than control KFs, while PLK4‐overexpressing KFs exhibited higher levels of the cleavage products of caspase‐3 and caspase‐9 than control NFs (Figure 4D). In summary, these data suggest that PLK4 inhibits KF apoptosis by inhibiting the caspase‐9/3 signaling pathway.

### 2.5 High PLK4 expression may decrease the percentage of KFs at the G0/G1 phase via the p53/p21/Cyclin D1 pathway

**FIGURE 5:**
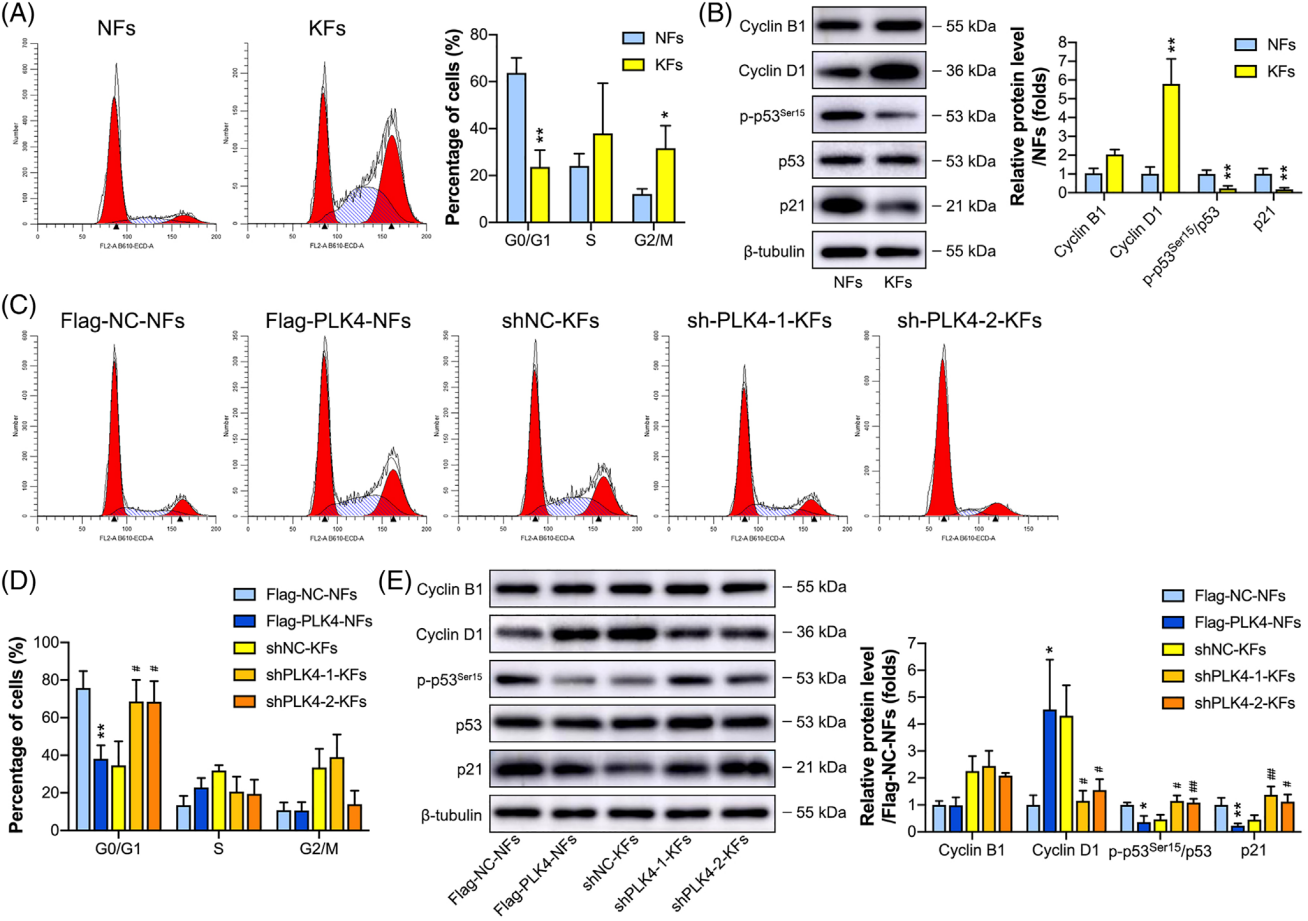
PLK4 induces cell cycle arrest at the G0/G1 phase in KFs. (A) *Left*, Flow cytometry analysis of the cell cycle using propidium (PI) labeling in NFs and KFs, and representative images are shown (*n* = 3 donors). *Right*, Quantitative analysis of the percentages of cells in the G0/G1, S, and G2/M phases of the cell cycle (*n* = 3 donors; *p < 0.05, **p < 0.01, compared with NFs). (B) *Left*, Western blot analysis of cell cycle‐related proteins, including Cyclin B1, Cyclin D1, p‐p53^Ser15^, p53, and p21, in NFs and KFs, and representative images are shown (*n* = 3 donors). *Right*, Quantitation of cell cycle‐related protein levels by densitometric analysis using ImageJ (*n* = 3 donors; **p < 0.01, compared with NFs). (C) Flow cytometric analysis of the cell cycles of the Flag‐NC‐NFs, Flag‐PLK4‐NFs, shNC‐KFs, shPLK4‐1‐KFs, and shPLK4‐2‐KFs using PI labeling, and representative images are shown (*n* = 3 donors). (D) Quantitative analysis of the percentages of cells in the G0/G1, S, or G2/M phases of the cell cycle (*n* = 3 donors; **p < 0.01, compared with Flag‐NC‐NFs; #*p* < 0.05, compared with shNC‐KFs). (E) *Left*, Western blot analysis of Cyclin B1, Cyclin D1, p‐p53^Ser15^, p53, and p21 in Flag‐NC‐NFs, Flag‐PLK4‐NFs, shNC‐KFs, shPLK4‐1‐KFs, and shPLK4‐2‐KFs, and representative images are shown (*n* = 3 donors). *Right*, Quantitation of cell cycle‐related protein levels by densitometric analysis using ImageJ (*n* = 3 donors; *p < 0.05, **p < 0.01, compared with Flag‐NC‐NFs; #*p* < 0.05, ##*p* < 0.01, compared with shNC‐KFs). KFs, keloid fibroblasts; NFs, normal skin fibroblasts; PLK4, Polo‐like kinase 4; shRNAs, short hairpin RNAs; shNC, negative control shRNA.

Flow cytometry was performed to analyze the cell cycle distribution in KFs and NFs. As shown in Figure 5A, KFs had a lower percentage of cells in the G0/G1 phase but a higher percentage of cells in the G2/M phase than NFs, and no significant difference in the percentage of cells in the S phase was observed between the two groups. Next, to explore the mechanism underlying cell cycle regulation in KFs, we examined the expression of important cell cycle regulators in KFs and NFs by western blot analysis (Figure 5B). The expression level of Cyclin B1, a G2/M‐phase control cyclin, was slightly higher in KFs than in NFs, but the difference was not significant. However, the expression of the Cyclin D1 protein, which positively controls the progression of the G1 to S phase transition,^31^ was significantly elevated in KFs compared to NFs. Because p53 was reported to be negatively regulated by PLK4,^32^, ^33^ we examined the levels of phosphorylated p53^Ser15^ and total p53, revealing no significant difference in the levels of total p53 between KFs and NFs. However, the level of phosphorylated p53^Ser15^ was downregulated in KFs compared to NFs. We further determined whether the downregulation of p53^Ser15^ phosphorylation affected the protein level of p21, a downstream effector and transcriptional target of p53 and an important cell cycle inhibitor. Western blot analysis revealed that the expression of the p21 protein was also decreased in KFs. These results indicated that the high expression of PLK4 in KFs may promote the G1 to S cell cycle transition and thus decrease the percentage of cells at the G0/G1 phase via the p53/p21/Cyclin D1 pathway.

To further validate this hypothesis, we examined the cell cycle phase distributions in the PLK4 knockdown and overexpression cell lines, including shNC‐KFs, shPLK4‐1‐KFs, shPLK4‐2‐KFs, Flag‐NC‐NFs, and Flag‐PLK4‐NFs, by flow cytometry (Figure 5C). The percentages of shPLK4‐1‐KFs and shPLK4‐2‐KFs in the G0/G1 phase were significantly higher than those of shNC‐KFs, while the percentage of Flag‐PLK4‐NFs in the G0/G1 phase was significantly lower than that of Flag‐NC‐NFs. However, knockdown or overexpression of PLK4 did not affect the percentages of transfected KFs and NFs in the G2/M phase (Figure 5D). Western blot analysis of cell cycle‐related markers yielded similar results (Figure 5E). Knockdown of PLK4 expression did not affect Cyclin B1 expression but decreased Cyclin D1 expression and increased the accumulation of the phosphorylated p53^Ser15^ and p21 proteins in KFs. In contrast, overexpression of PLK4 did not affect Cyclin B1 expression but showed the opposite effect on Cyclin D1, phosphorylated p53^Ser15^, and p21 expression in NFs (Figure 5E).

### 2.6 Inhibition of PLK4 by Cen‐B reduces the proliferation, migration, and invasion of KFs

**FIGURE 6:**
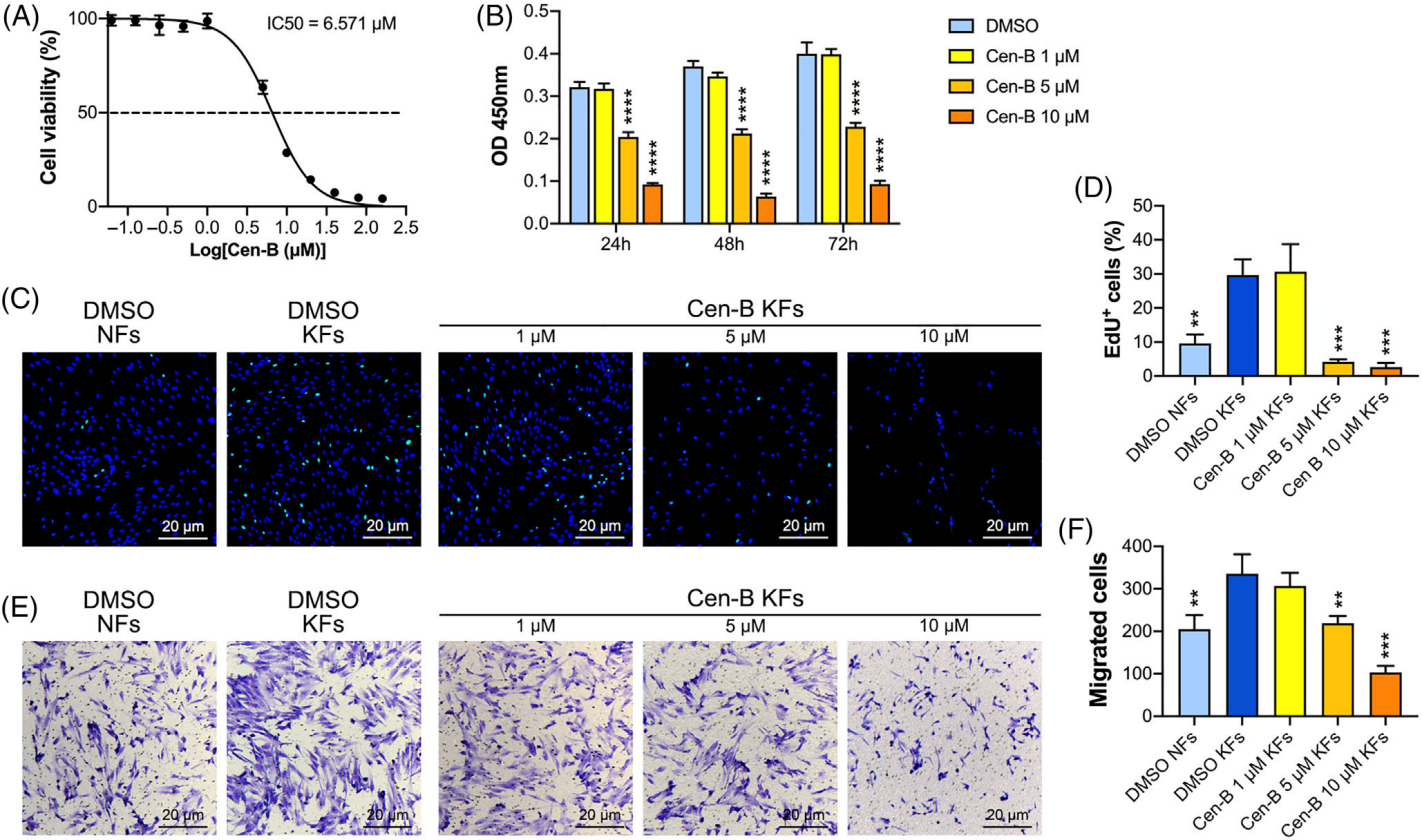
Cen‐B reduces the proliferation, migration, and invasion of human KFs. (A) The IC50 curve for Cen‐B in KFs Was calculated by the CCK‐8 assay (*n* = 3 donors). (B) KFs were treated with different concentrations of Cen‐B for 24, 48, and 72 h, and cell viability was measured by the CCK‐8 assay (*n* = 3 donors; ****p < 0.0001, compared with dimethyl sulfoxide [DMSO]‐treated KFs). (C) EdU and Hoechst dual‐labeling of NFs and KFs after 48 h of treatment with DMSO or Cen‐B at different concentrations. Representative images of EdU^+^ cells (*n* = 3 donors). (D) Quantitative analysis of EdU^+^ cells (*n* = 3 donors; **p < 0.01, ***p < 0.001, compared with DMSO‐treated KFs). (E) Cell invasion assays of KFs and NFs on 8‐μm pore size filters were performed after 48 h of treatment with DMSO or Cen‐B at different concentrations, and representative images of the migrated cells are shown (*n* = 3 donors). (F) Quantitative analysis of the migrated cells (*n* = 3 donors; **p < 0.01, ***p < 0.001, compared with DMSO‐treated KFs). CCK‐8, Cell Counting Kit‐8; Cen‐B, Centrinone B; EdU, 5‐ethynyl‐2′‐deoxyuridine; KFs, keloid fibroblasts.

Cen‐B, a highly specific inhibitor of PLK4, was utilized to inhibit the activity of PLK4 in KFs. Cen‐B exhibited inhibitory activity on PLK4 with a half‐maximal inhibitory concentration (IC_50_) value of 6.571 μM in KFs (Figure 6A). Cen‐B at concentrations of 1, 5, and 10 μM was used in subsequent experiments. As shown in Figure 6B, the CCK‐8 assay demonstrated that Cen‐B markedly decreased cell proliferation in a dose‐ and time‐dependent manner. Furthermore, the 5‐ethynyl‐2′‐deoxyuridine (EdU) incorporation assay demonstrated that 48 h of Cen‐B treatment potently decreased the percentages of EdU^+^ cells in KFs in a concentration‐dependent manner (Figure 6C,E). Similar results were also observed in the Transwell invasion assay, as the invasion capacity of KFs was significantly inhibited in a concentration‐dependent manner after 48 h of exposure to Cen‐B (Figure 6D,F). Taken together, these results demonstrated that Cen‐B suppresses the PLK4‐mediated proliferation, migration, and invasion of KFs.

### 2.7 Inhibition of PLK4 with Cen‐B induces apoptosis and G0/G1‐phase cell cycle arrest in KFs

**FIGURE 7:**
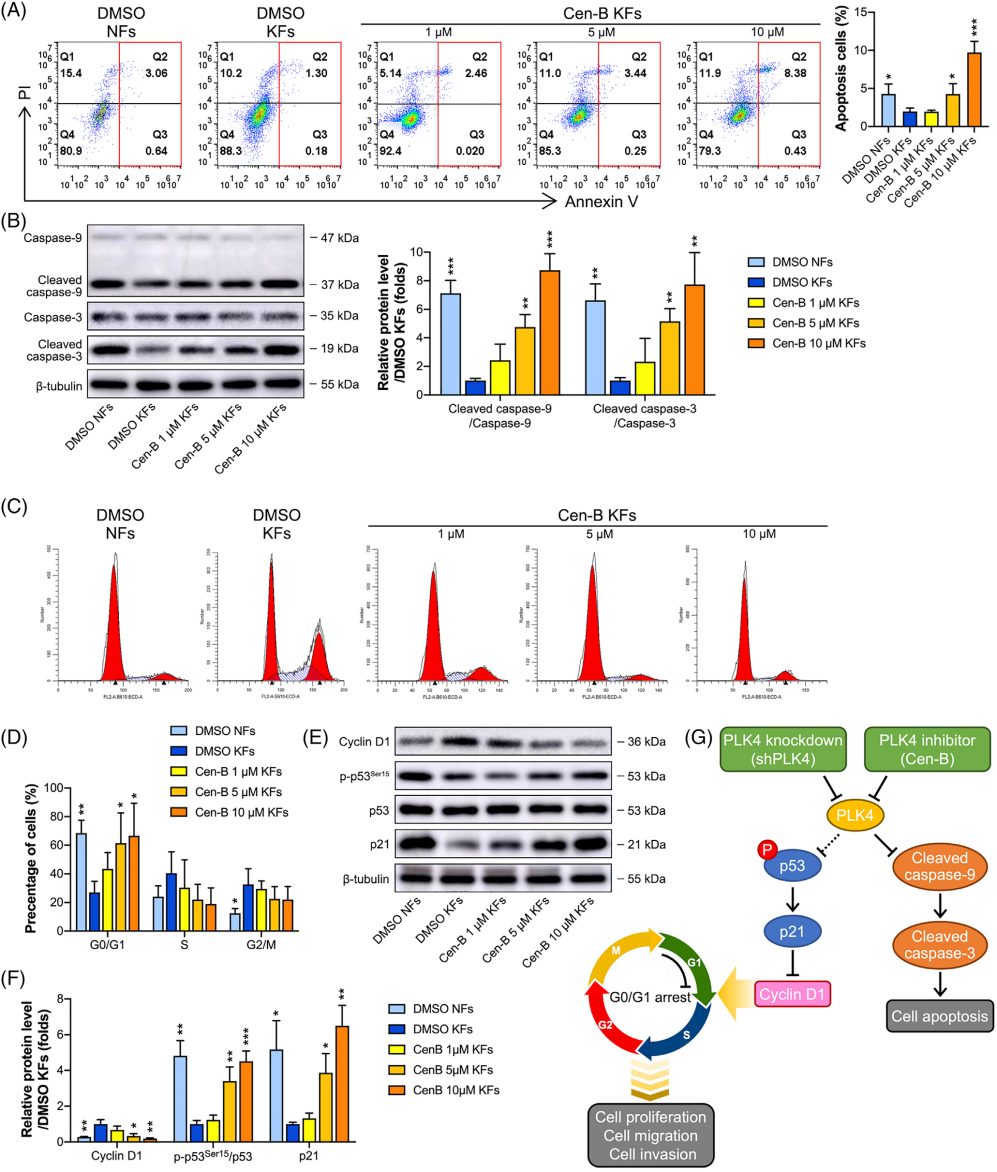
Cen‐B induces apoptosis and cell cycle arrest in KFs. (A) *Left*, KFs and NFs were treated with dimethyl sulfoxide (DMSO) or different concentrations of Cen‐B for 48 h, and cell apoptosis was detected by flow cytometry after annexin V and propidium (PI) dual‐labeling, and representative images of the apoptotic cells are shown (*n* = 3 donors). *Right*, Quantitative analysis of apoptotic cells in both the early and late phases (*n* = 3 donors; *p < 0.05, ***p < 0.001, compared with DMSO‐treated KFs). (B) *Left*, Western blot analysis of caspase‐9, cleaved caspase‐9, caspase‐3, and cleaved caspase‐3 expression in NFs and KFs after 48 h of treatment with DMSO or Cen‐B, and representative images are shown (*n* = 3 donors). *Right*, Quantitation of apoptosis‐related protein levels by densitometric analysis using ImageJ (*n* = 3 donors; **p < 0.01, ***p < 0.001, compared with DMSO‐treated KFs). (C) KFs and NFs were treated with DMSO or different concentrations of Cen‐B for 48 h, and the cell cycle was detected by flow cytometry after PI dual‐labeling. Representative images are shown (*n* = 3 donors). (D) Quantitative analysis of the percentages of cells in the G0/G1, S, and G2/M phases of the cell cycle (*n* = 3 donors; *p < 0.05, **p < 0.01, compared with DMSO‐treated KFs). (E) KFs and NFs were treated with DMSO or different concentrations of Cen‐B for 48 h, and western blot analysis of Cyclin D1, p‐p53^Ser15^, p53, and p21 was performed. Representative images are shown (*n* = 3 donors). (F) Quantitation of cell cycle‐related protein levels by densitometry analysis using ImageJ (*n* = 3 donors; *P < 0.05, **P < 0.01, ***P < 0.001, compared with DMSO‐treated KFs). (G) Schematic demonstrating the possible mechanism of G0/G1 arrest and apoptosis induced by PLK4 knockdown and inhibition in KFs. Cen‐B, Centrinone B; KFs, keloid fibroblasts; NFs, normal skin fibroblasts.

We then explored the functions of Cen‐B in cell apoptosis and cell cycle regulation. The KFs were treated with dimethyl sulfoxide (DMSO) or different Cen‐B concentrations for 48 h. Flow cytometry analysis demonstrated that the Cen‐B treatment had a positive effect on the percentages of apoptotic cells, which was significantly enhanced as the concentration increased (Figure 7A). Western blot analysis of apoptosis‐related proteins revealed that when the Cen‐B concentration increased, the expression levels of cleaved caspase‐9/caspase‐9 and cleaved caspase‐3/caspase‐3 were significantly elevated (Figure 7B). These data substantiated that Cen‐B‐induced cell death is driven, at least in part, by caspase‐9/3‐mediated apoptosis.

In addition, flow cytometry analysis demonstrated that the Cen‐B treatment significantly increased the percentage of cells in the G0/G1 phase in a concentration‐dependent manner and had no effect on the percentages of cells in the S and G2/M phases (Figure 7C,D). Furthermore, Cen‐B treatment decreased the protein level of Cyclin D1 and increased the protein levels of phosphorylated p53^Ser15^ and p21, and these effects were consistent with those observed in the flow cytometric analysis (Figure 7E,F). These data suggested that Cen‐B treatment‐induced cell cycle arrest at the G0/G1 phase may be via the p53/p21/Cyclin D1 pathway. The possible underlying mechanism of PLK4‐mediated apoptosis and cell cycle regulation in KFs is shown in Figure 7G.

## 3 DISCUSSION

Keloids represent benign fibroproliferative skin tumors that display many cancer‐like characteristics, such as progressive uncontrolled growth, lack of spontaneous regression, and extremely high rates of recurrence. PLK4 has been identified as a causative factor for the development of various tumors^13^, ^14^, ^15^, ^16^, ^17^, ^18^ and proliferative tissues.^19^, ^20^, ^21^ However, to the best of our knowledge, no study has investigated its role in keloids. The present study indicated for the first time that PLK4 is aberrantly upregulated in the dermal tissues of quasi‐neoplastic keloids. Stepwise investigation revealed that PLK4 upregulation enhanced the proliferation, migration, and invasion of KFs. Furthermore, inhibition of PLK4 with Cen‐B promoted cell apoptosis via the caspase‐9/3 pathway and induced cell cycle arrest at the G0/G1 phase via the p53/p21/Cyclin D1 pathway in KFs. These findings suggested that inhibition of PLK4 with Cen‐B may be a novel therapeutic strategy for keloid patients.

Although PLK4 was shown to be aberrantly expressed in many tumor types in the recently published literature, it is also expressed at low levels in proliferative tissues, such as the pancreas and skin.^20^, ^21^ In the present study, we found that keloids, which are considered dermal fibroproliferative tumors, exhibited higher mRNA and protein levels of PLK4 in the dermal layer, especially in the superficial dermis, than in their corresponding adjacent normal skin tissues. Although high levels of PLK4 expression were also found in the epidermal layer of keloids and corresponding adjacent normal skin tissues, the differences were not significant, and the high level of PLK4 expression in the epidermis may have been caused by the high rate of physiological cell renewal in the epidermal tissues of both keloids and normal skin. Furthermore, at the cell level, we observed that PLK4 expression was in the nucleoli and cytoplasm of both KFs and NFs. The location of PLK4 expression remains controversial in the literature. PLK4 expression was initially reported to be restricted to the centrosomes.^34^, ^35^ While recent studies observed that PLK4 was also expressed outside of the centrosomes including nucleoli, cleavage furrow of dividing cells, and protrusions.^11^, ^36^ Our results shthat PLK4 was expressed in the nucleoli and cytoplasm of both KFs and NFs may suggest a different function unrelated to centrosomes. In addition, although high expression of PLK4 has been linked with an aberrant centrosome number in several cancers,^16^, ^24^, ^37^ the high expression of PLK4 did not result in abnormal centrosome amplification in KFs in this study. The proper number of centrosomes in cells is regulated by a complex network.^38^, ^39^ PLK4 activity is required for centrosome amplification that can initiate tumorigenesis and has been considered a hallmark of cancer.^40^ However, keloids are quasi‐neoplastic lesions and essentially not malignant tumors, and KFs may have normal centrosome duplication.

Accumulating evidence has demonstrated a link between hyperproliferative fibroblasts and neoplastic aggression in cancers, such as breast cancer^22^ and neuroblastoma.^41^ Keloids are considered quasi‐neoplastic lesions that are characterized by the abnormal proliferation and invasive growth of dermal fibroblasts. PLK4 is an important cell cycle regulatory protein that is involved in mitotic progression and subsequent cell proliferation. Thus, we speculated that PLK4 may also be involved in the aggressive behavior of keloids. In this study, we first established a PLK4 knockdown model in KFs and a PLK4 overexpression model in NFs. The results presented herein indicated that elevated PLK4 expression was essential for the proliferation, migration, and invasion of KFs. Knockdown of PLK4 expression in KFs inhibited their proliferation, migration, and invasion. Conversely, overexpression of PLK4 enhanced the proliferation, migration, and invasion capabilities of NFs. These findings were following those of previous reports indicating that upregulation of PLK4 expression played a pivotal role in the cell viability, proliferation, migration, invasion, tumorigenesis, and progression of several tumors.^42^, ^43^ Nevertheless, the cell growth was not inactivated by mitotic inhibitors in the cell migration/invasion assays, although restricted control groups were employed in these experiments, the enhanced cell migration in the scratches and cell invasion in the chambers may be caused by a combined effect of enhanced cell proliferation and migration.

An imbalance between fibroblast proliferation and apoptosis is thought to be the cytological basis for the continuous proliferation of keloid cells.^2^ We first confirmed that KFs had lower rates of apoptosis than NFs and further found that the low apoptosis rate was related to the high level of PLK4 expression in KFs. Recent studies have shown that PLK4 is an important antiapoptotic molecule in cancer cells.^20^, ^44^ Tian et al.^45^ reported that the downregulation of PLK4 expression facilitated cell apoptosis by increasing the expression of caspase‐3 in neuroblastoma cells. Furthermore, the increased kinase activity of PLK4 in cancer cells suppressed caspase‐9‐ and caspase‐3‐mediated apoptosis, whereas decreased expression of PLK4 potentiated apoptosis.^46^ Similar results were obtained in this study. We showed that PLK4 knockdown by shRNA in KFs augmented the expression levels of cleaved caspase‐9 and cleaved caspase‐3, which are hallmarks of apoptosis, while PLK4 overexpression in NFs decreased cleaved caspase‐9/3 expression and suppressed caspase‐9/3‐mediated apoptosis.

One of the most distinguishing hallmarks of keloids is cell cycle aberrations that lead to the hyperproliferation of KFs.^47^ Our results were consistent with those of studies reporting that KFs exhibited a higher percentage of cells distributed in the G2/M phase than NFs. Moreover, we also observed that the KFs had a lower percentage of cells distributed in the G0/G1 phase than the NFs. Interestingly, knockdown of PLK4 expression in KFs induced cell cycle arrest at the G0/G1 phase but did not influence the G2/M phase. The induction of G0/G1 cell cycle arrest has been frequently observed in various studies under different treatment conditions for tumor therapy, such as X‐ray radiation,^48^ small‐molecule treatment,^49^, ^50^ and signaling pathway blockade.^29^, ^51^ However, the mechanism underlying the activation of the cell cycle‐related pathway differs among studies. PLK4 reportedly mediates G0/G1 cell cycle arrest in a p53‐dependent manner.^17^, ^52^, ^53^ In addition, p53‐dependent cell cycle arrest is mainly mediated by p21.^54^, ^55^ Our data also demonstrated that the G0/G1 cell cycle arrest in KFs induced by PLK4 knockdown was triggered in a p53/p21/Cyclin D1‐dependent manner, which was consistent with the results observed in several other cancer studies.^17^, ^52^, ^53^

As a critical regulator of cancer development, PLK4 has emerged as a therapeutic target for multiple cancers.^42^, ^43^, ^56^ PLK4 inhibitors, such as CFI‐400945,^15^ centrinone,^57^, ^58^ Cen‐B,^27^, ^59^ YLZ‐F5,^26^ and YLT‐11,^25^ have shown anticancer effects in multiple cancers. In this study, Cen‐B, a potent and highly selective PLK4 inhibitor,^60^ was used to inhibit the activity of PLK4 in KFs for the first time. Consistent with the effects of PLK4 knockdown, Cen‐B treatment significantly inhibited various pathological phenotypes of KFs, including their proliferation, migration, and invasion in vitro, in a dose‐ and time‐dependent manner. Furthermore, Cen‐B treatment induced apoptosis via the caspase‐9/3 pathway and induced cell cycle arrest at the G0/G1 phase via the p53/p21/Cyclin D1 pathway in KFs. Our observations indicate the potential clinical application of Cen‐B for keloid therapy, although the therapeutic activity and safety may need to be further investigated in animal models.

In summary, we reported herein that keloids expressed high levels of PLK4, which induced tumor‐like malignant biological behaviors in KFs, including enhanced proliferation, migration, and invasion. Furthermore, knockdown of PLK4 expression or inhibition of PLK4 activity with Cen‐B induced apoptosis via the caspase‐9/3 pathway, and the induced cell cycle arrest at the G0/G1 phase may be via the p53/p21/Cyclin D1 pathway. These findings highlight the importance of PLK4 as a promising therapeutic target for keloids.

## 4 MATERIALS AND METHODS

### 4.1 Patients and tissue samples

Keloid and adjacent normal skin tissue samples (Figure 1A) were harvested from 21 patients undergoing keloid resection surgery at the Department of Plastic and Reconstructive Surgery, Shanghai Ninth People's Hospital, Shanghai Jiao Tong University School of Medicine, and written consent was obtained preoperatively. The study protocol was approved by the ethics committee of our institute (ethics number: SH9H‐2021‐A1042‐SB). Keloids were diagnosed clinically and distinguished from hypertrophic scars according to the presence of invasive growth beyond the original wound border. No keloid patients had received scar treatment. The detailed information of the 21 patients is shown in Table 1.

**TABLE 1:** Detailed demographic information and applications of the keloid and their adjacent normal skin samples

Donor no.	Age, years	Gender	Location	Applications
1	21	M	Thoraxz	(1) WB analysis of PLK4 protein expression. (2) IHC analysis of PLK4 protein expression. (3) RT‐PCR analysis of PLK4 mRNA expression. (4) IF analysis of PLK4 and γ‐tubulin foci proteins expression. (5) FC analysis of apoptosis and cell cycle.
2	36	M	Thorax	(1) WB analysis of PLK4 protein expression. (2) IHC analysis of PLK4 protein expression. (3) RT‐PCR analysis of PLK4 mRNA expression. (4) IF analysis of PLK4 and γ‐tubulin foci proteins expression. (5) FC analysis of apoptosis and cell cycle.
3	35	M	Thorax	(1) WB analysis of PLK4 protein expression. (2) IHC analysis of PLK4 protein expression. (3) RT‐PCR analysis of PLK4 mRNA expression. (4) IF analysis of PLK4 and γ‐tubulin foci proteins expression. (5) FC analysis of apoptosis and cell cycle.
4	27	F	Shoulder/back	(1) WB analysis of PLK4 protein expression. (2) IHC analysis of PLK4 protein expression. (3) RT‐PCR analysis of PLK4 mRNA expression. (4) IF analysis of PLK4 and γ‐tubulin foci proteins expression. (5) FC analysis of apoptosis and cell cycle.
5	30	F	Ear	(1) WB analysis of PLK4 protein expression. (2) IHC analysis of PLK4 protein expression. (3) RT‐PCR analysis of PLK4 mRNA expression. (4) IF analysis of PLK4 and γ‐tubulin foci proteins expression. (5) FC analysis of apoptosis and cell cycle.
6	33	F	Thorax	(1) WB analysis of PLK4 protein expression. (2) IHC analysis of PLK4 protein expression. (3) RT‐PCR analysis of PLK4 mRNA expression. (4) IF analysis of PLK4 and γ‐tubulin foci proteins expression. (5) FC analysis of apoptosis and cell cycle.
7	22	F	Ear	(1) WB analysis of PLK4 protein expression. (2) RT‐PCR analysis of PLK4 mRNA expression. (3) Establishment of PLK4 knockdown and overexpression cell lines.
8	25	F	Thorax	(1) WB analysis of PLK4 protein expression. (2) RT‐PCR analysis of PLK4 mRNA expression. (3) Establishment of PLK4 knockdown and overexpression cell lines.
9	12	M	Shoulder/back	(1) WB analysis of PLK4 protein expression. (2) RT‐PCR analysis of PLK4 mRNA expression. (3) Establishment of PLK4 knockdown and overexpression cell lines.
10	22	F	Shoulder/back	(1) WB analysis of PLK4 protein expression. (2) RT‐PCR analysis of PLK4 mRNA expression. (3) Cen‐B treatment.
11	54	F	Shoulder/back	(1) WB analysis of PLK4 protein expression. (2) RT‐PCR analysis of PLK4 mRNA expression. (3) Cen‐B treatment.
12	22	F	Thorax	(1) WB analysis of PLK4 protein expression. (2) RT‐PCR analysis of PLK4 mRNA expression. (3) Cen‐B treatment.
13	24	F	Thorax	(1) WB analysis of PLK4 protein expression. (2) RT‐PCR analysis of PLK4 mRNA expression.
14	32	F	Ear	(1) WB analysis of PLK4 protein expression. (2) RT‐PCR analysis of PLK4 mRNA expression.
15	23	F	Thorax	(1) WB analysis of PLK4 protein expression. (2) RT‐PCR analysis of PLK4 mRNA expression.
16	48	M	Thorax	(1) WB analysis of PLK4 protein expression. (2) RT‐PCR analysis of PLK4 mRNA expression.
17	28	M	Ear	(1) WB analysis of PLK4 protein expression. (2) RT‐PCR analysis of PLK4 mRNA expression.
18	42	M	Abdomen	(1) WB analysis of PLK4 protein expression. (2) RT‐PCR analysis of PLK4 mRNA expression.
19	22	F	Ear	(1) WB analysis of PLK4 protein expression. (2) RT‐PCR analysis of PLK4 mRNA expression.
20	30	M	Thorax	(1) WB analysis of PLK4 protein expression. (2) RT‐PCR analysis of PLK4 mRNA expression.
21	22	F	Ear	(1) WB analysis of PLK4 protein expression. (2) RT‐PCR analysis of PLK4 mRNA expression.

Abbreviations: Cen‐B, centrinone B; F, female; FC, flow cytometry; IF, immunofluorescence; IHC, immunohistochemistry; M, male; RT‐PCR, real‐time polymerase chain reaction; WB, western blot.

### 4.2 Isolation and culture of cells

KFs and NFs were derived from the keloid dermis and the adjacent normal skin dermis from the same patients as previously described.^61^ Briefly, the specimens were sectioned into 2–3‐cm pieces and washed with ice‐cold phosphate‐buffered saline (PBS). The specimens were then incubated with 3 mg/ml Dispase II (Roche, Switzerland) in Dulbecco's modified Eagle's medium (DMEM; Gibco, USA) at 37°C for 1 h. The epidermis was removed, and the dermis was minced into small pieces, followed by enzymatic digestion with 1 mg/ml collagenase I (Nordmark, Germany) in DMEM at 37°C for 2.5 h. After digestion, the cell suspension was filtered through a 70‐μm cell strainer and centrifuged. The supernatant was discarded, and the cells were cultured in a complete medium (DMEM supplemented with 10% fetal bovine serum (FBS), and 1% penicillin–streptomycin [all from Gibco]).

### 4.3 Plasmid construction and transfection

shRNAs targeting human *PLK4* were subcloned into the PGMLV lentiviral vector (Genomeditech). The primers for the construction of PLK4 knockdown vectors and full‐length *PLK4* cDNA for the construction of the PLK4 overexpression vector are shown in Table S1. The PCR product and PGMLV‐3 × Flag vector (Genomeditech) were used to construct the overexpression vector (Flag‐PLK4), while the empty pLVX‐vector (Flag‐NC) was used as a control. For lentiviral packaging, 293T cells were seeded onto 10‐cm dishes and co‐transfected with plasmids using HG transgene reagent. Sixteen hours post‐transfection, the cells were transferred to a fresh complete medium and incubated for another 48 h. The lentiviral supernatants were collected, filtered, and added to primary KFs or NFs in the presence of polybrene. At 16 h post‐infection, the cells were selected by puromycin in a fresh complete medium for 7 days. The knockdown or overexpression efficiency was verified by real‐time PCR and western blotting (Figures 2B, 3B). Three biological replicates were performed for the establishment of PLK4 knockdown KF cell lines and PLK4 overexpression NF cell lines.

### 4.4 Treatment of cells

Cells were seeded at a density of 6 × 10^4^ cells/cm^2^ and cultured in a complete medium. After reaching 80% confluence, the cells were serum‐starved for 12 h and then treated with Cen‐B (Tocris Biosciences, UK) at concentrations of 1, 5, and 10 μM for the indicated amounts of time, while the KFs and NFs derived from the same patients treated with DMSO (diluted with DMEM containing 10% FBS) served as positive and negative controls, respectively. Three biological replicates were performed for Cen‐B treatment and used in the following experiments.

### 4.5 Scratch wound migration assay

Cells were seeded onto six‐well plates at a density of 6 × 10^4^ cells/cm^2^ and cultured in a complete medium overnight. The cell monolayer was scratched with a 200‐μL pipette tip and then cultured in DMEM supplemented with 0.5% FBS. Five random fields within the scratched area were captured at 0, 12, and 24 h after treatment. The cell migration area was calculated by ImageJ software. Three biological replicates were performed for the scratch wound migration assay.

### 4.6 Transwell invasion assay

A total of 2.5 × 10^4^ cells were suspended in 200 μl of serum‐free DMEM and plated on the top compartment of a Transwell chamber with 8‐μm pores (Corning, USA). Then, 600 μl of complete medium was added to the bottom compartment. After 24 h, the migrated cells were fixed and stained with 0.5% crystal violet. Cells from five random fields were captured using a microscope. Three biological replicates were performed for the Transwell invasion assay.

### 4.7 Cell protrusion assay

A total of 5 × 10^4^ cells were suspended in 200 μl of serum‐free DMEM and plated on the top compartment of a Millicell insert with 1‐μm pores (Millipore, USA). Then, 600 μl of complete medium was added to the bottom compartment. After 10 h, the cells were fixed, permeabilized, and blocked. Finally, the cells were double‐stained with phalloidin (1:400; Abcam, Cambridge, UK) and Hoechst (1:800; RiboBio, Guangzhou, China). The filters were visualized using a laser confocal microscope (Leica, Germany). Three biological replicates were performed for the cell protrusion assay.

### 4.8 CCK‐8 assay

According to the cell counting results, different amounts of cells were seeded in a 96‐well plate (1562, 3125, 6250, 12,500, 18,750, 25,000, 37,500, and 50,000 cells per well). Six wells were used for each group, and the CCK‐8 standard curve was fitted according to the measurement results. Moreover, cells were seeded in 96‐well plates at a density of 3000 cells per well and cultured for 0, 24, 48, 72, and 96 h with a complete medium. Then, the cells were incubated with a CCK‐8 reagent (Dojindo, Japan) for 2 h at 37°C. Thereafter, the medium was harvested to measure the optical density values at 450 nm using a microplate reader (Thermo, Finland). The absolute number of cells was calculated by standard curve and the cell proliferation curve was plotted. Three biological replicates were performed for the CCK‐8 assay.

### 4.9 EdU assay

Cells were stained with EdU (RiboBio) for 8 h. EdU^+^ cells (green) and Hoechst^+^ cells (blue) were imaged. The EdU incorporation rate was calculated as the ratio of the number of EdU^+^ cells to the number of Hoechst^+^ cells. A total of 5 fields of view were selected randomly to calculate the positive rate. The data were analyzed based on at least three biological replicates.

### 4.10 Flow cytometric analysis

KFs, shNC‐KFs, shPLK4‐1‐KFs, shPLK4‐2‐KFs, NFs, Flag‐NC‐NFs, and Flag‐PLK4‐NFs were collected. For apoptosis analysis, the cells were stained with annexin V and propidium iodide (PI) (BD Biosciences, USA) and detected by flow cytometry. The data were analyzed using FlowJo software (Treestar, USA). For cell cycle analysis, cells were fixed with 75% ethanol, suspended in PI solution (50 μg/ml), and incubated for 15 min. Then, the cells were detected by flow cytometry, and the data were analyzed using ModFit LT software (Verity Software, USA). KFs and NFs were derived from the same patients, and the data were analyzed based on at least three biological replicates.

### 4.11 RNA extraction and real‐time PCR

Cells were washed with PBS and lysed with a Multisource Total RNA Miniprep Kit (Axygen, USA) according to the manufacturer's protocol. Then, reverse transcription was performed using a cDNA Reverse Transcription Kit (Takara, Japan) and cDNA was subjected to real‐time PCR using the PrimeScript RT‐PCR Kit (Takara). The PCR primers are provided in Table S2. The comparison of *PLK4* expression between keloid dermis and adjacent normal skin dermis was made based on 21 biological replicates, while the knockdown or overexpression efficiency was verified based on three biological replicates.

### 4.12 Histology and immunostaining assays

Cells were fixed and permeabilized with 0.1% Triton X‐100. The cells were blocked with 5% bovine serum albumin (BSA) and then immunostained with rabbit anti‐PLK4 antibody (1:200; Abcam), mouse anti‐Ki67 antibody (1:200; Abcam), or mouse anti‐γ‐tubulin antibody (1:200; Sigma‐Aldrich, USA), followed by incubation with a goat anti‐rabbit or goat anti‐mouse secondary antibody (1:400; Invitrogen), phalloidin (1:400; Abcam), and 4',6‐diamidino‐2‐phenylindole (1:800; Sigma‐Aldrich). Samples were mounted with an anti‐fade reagent (Cell Signaling Technology, USA). Cell area analysis was performed using ImageJ software. The relative cell areas were normalized to NFs. The immunofluorescence staining of KFs and NFs was conducted on at least three biological replicates.

Tissue samples were fixed with 4% paraformaldehyde and embedded in paraffin. The sections were blocked with 5% BSA and incubated with a rabbit anti‐PLK4 antibody (1:200; Abcam). After sequential incubation with a biotinylated secondary antibody (Dako, Denmark) and an ABC‐alkaline phosphatase complex (Vector, USA), specific staining was revealed by using Fast Red (Dako). Immunohistochemistry staining of keloid and adjacent normal skin samples was conducted on at least three biological replicates.

### 4.13 Western blot analysis

Proteins were extracted with Radio Immunoprecipitation Assay lysis buffer containing 1 mM phenylmethylsulfonyl fluoride (Beyotime, China). Protein samples were separated by sodium dodecyl sulfate‐polyacrylamide gel electrophoresis and subjected to western blot analysis using the following primary antibodies: anti‐PLK4 (1:500; Abcam), anti‐p53 (1:1000), anti‐phospho‐p53^Ser15^ (1:1000), anti‐Cyclin B1 (1:1000), anti‐Cyclin D1 (1:1000), anti‐caspase‐3 (1:1000), anti‐cleaved caspase‐3 (1:1000), anti‐caspase‐9 (1:1000), anti‐cleaved caspase‐9 (1:1000), and anti‐β‐tubulin (1:1000). Then, the membranes were incubated with secondary antibodies (1:1000; all from Cell Signaling Technology). The protein bands were eventually visualized using an enhanced chemiluminescence detection kit (Millipore). The comparison of PLK4 expression between keloid dermis and adjacent normal skin dermis was made based on 21 biological replicates, while other western blot assays were conducted on at least three biological replicates.

### 4.14 Statistical analysis

Statistical analysis was performed with GraphPad Prism software version 9.0 (GraphPad Software, USA). The data are presented as the mean ± SD of at least three biological replicates. Student's *t*‐test was used to determine the significance between the two groups. Standard Pearson correlation analysis was performed to explore the correlation of the relative MFI of PLK4 with the percentage of Ki67^+^ cells. Pearson's coefficients with *p* values were calculated. A *p*‐value of <0.05 was considered to indicate statistical significance.

## AUTHOR CONTRIBUTIONS

**Ru‐Lin Huang**: Conceptualization, methodology, formal analysis, writing—review and editing, visualization, supervision, project administration, funding acquisition. **Chuanqi Liu**: Conceptualization, methodology, validation, investigation, resources, data curation, writing—review and editing, visualization. **Rao Fu**: Methodology, investigation, resources, investigation, data curation. **Yuxin Yan**: Methodology, resources, investigation, data curation. **Jing Yang**: Resources, investigation, data curation. **Xinggang Wang**: Conceptualization, resources, investigation, data curation. **Qingfeng Li**: Conceptualization, writing—review and editing, supervision, project administration, funding acquisition.

## FUNDING INFORMATION

This research was supported by grants from the National Natural Science Foundation of China (81871571) and the Shanghai Municipal Key Clinical Specialty (shslczdzk00901).

## CONFLICT OF INTEREST

The authors declare no conflict of interest.

## Supporting information


**Data S1** Supporting Information

## Data Availability

The data that support the findings of this study are available in the supplementary material of this article.
