# Supplementary material for: Downregulation of PLK4 expression induces apoptosis and G0/G1‐phase cell cycle arrest in keloid fibroblasts
Source: Cell Prolif. 2022 Jun 7;55(7):e13271. doi: 10.1111/cpr.13271 (PMC9251049; doi:10.1111/cpr.13271)
Supplement: Supplementary file 1 — Data S1 Supporting Information [file CPR-55-e13271-s001.docx]

# **Supplementary Tables**

# **Supplementary Table 1**

**Supplementary Table 1**. Vectors for PLK4 knockdown and overexpression

| Vectors | Sequence |
| --- | --- |
| *shPLK4-1* | 5’-GATCCGCAGAAGAAAGGCCACATTCTCTCGAGAGAATGTGGCCTTTCTTCTGCTTTTTT-3’ |
| *shPLK4-2* | 5’- GATCCGCTTCTGATAATGCACATTCTCTCGAGAGAATGTGCATTATCAGAAGCTTTTTT-3’ |
| *shPLK4-3* | 5’-GATCCGGTTACAAATGAAGGACTTGGCTCGAGCCAAGTCCTTCATTTGTAACCTTTTTT-3’ |
| *shNC* | 5’-GATCTGTTCTCCGAACGTGTCACGTTTCAAGAGAACGTGACACGTTCGGAGAATTTTTTC-3’ |
| *Flag-PLK4 cDNA* | F: 5’-GCGAATTCGAAGTATACCTCGAGGCCACCATGGCGACCTGCA-3’  R: 5’-GTCATGGTCTTTGTAGTCGGATCCATGAAAATTAGGAGTCGGATTAGAAAACATCA-3’ |

Abbreviations: F, forward primer; R, reverse primer.

# **Supplementary Table 2**

**Supplementary Table 2**. Primers for Real-Time PCR

| Gene | Sequence | Gene reference |
| --- | --- | --- |
| *PLK4* | F: 5’ -TTCTCGATACCTTCGTAGAGCTT-3’  R: 3’ -CTGAGTGACATCGTTCCATTGT-5’ | NM_001190801.2 |
| *ACTB* | F: 5’ -ACCAACTGGGACGACATGGAGAAA-3’  R: 3’ -TAGCACAGCCTGGATAGCAACGTA-5’ | NM_001101.5 |

Abbreviations: F, forward primer; R, reverse primer.

# **Supplementary Figures**

# **Supplementary Figure 1**


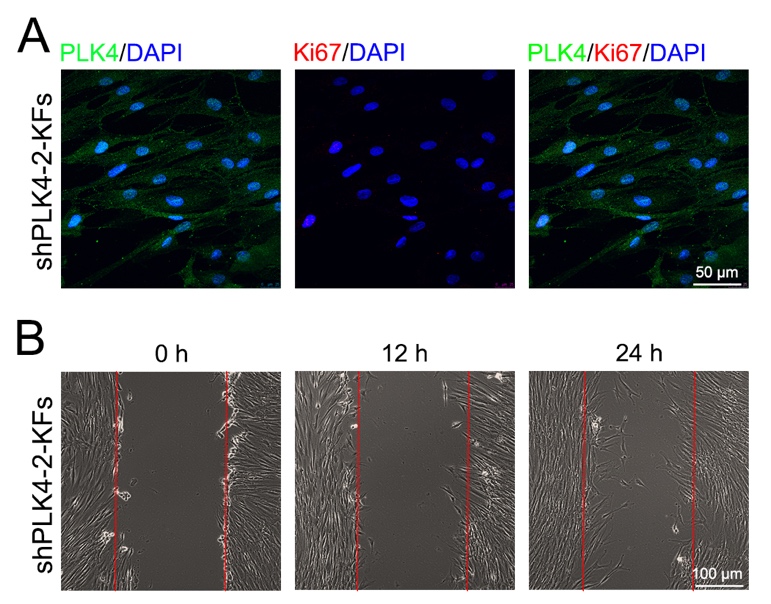


**Supplementary Figure 1. Knockdown of PLK4 by shPLK4-2 lentivirus inhibits KF proliferation and migration.**

(A) Immunofluorescence staining of PLK4 and Ki67 in shPLK4-2-KFs at 7 days post-transfection (n = 3 donors). (B) Scratch wound healing assays of shPLK4-2-KFs were performed at 7 days post-transfection, and representative images of the healed scratches at 0, 12, and 24 hours are shown (n = 3 donors).

# **Supplementary Figure 2**

**
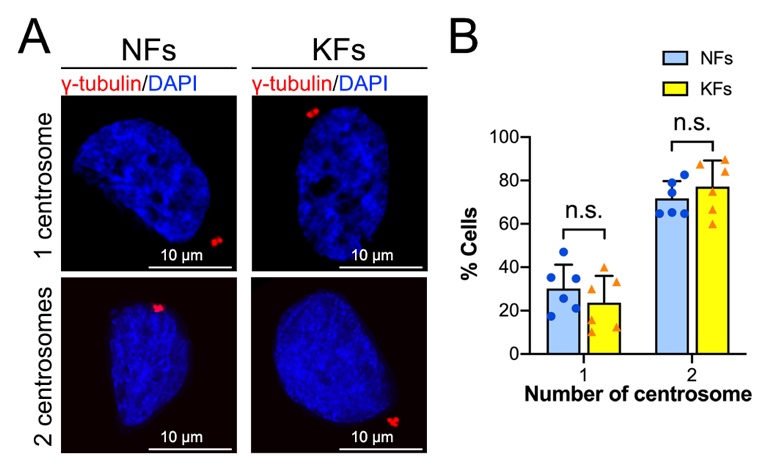
**

**Supplementary Figure 2. Centrosome number distribution in KFs and NFs**

(A) Immunofluorescence staining of γ-tubulin foci and DAPI in NFs and KFs (n = 6 donors). (B) Graph showing the centrosome number distribution in NFs and KFs (n = 6 donors, compared with NFs).
